# Relationships between gut microbiota, plasma metabolites, and metabolic syndrome traits in the METSIM cohort

**DOI:** 10.1186/s13059-017-1194-2

**Published:** 2017-04-13

**Authors:** Elin Org, Yuna Blum, Silva Kasela, Margarete Mehrabian, Johanna Kuusisto, Antti J. Kangas, Pasi Soininen, Zeneng Wang, Mika Ala-Korpela, Stanley L. Hazen, Markku Laakso, Aldons J. Lusis

**Affiliations:** 1 Department of Medicine, University of California, Los Angeles, Los Angeles, CA 90095 USA; 2 Estonian Genome Centre, University of Tartu, Tartu, 51010 Estonia; 3 Institute of Molecular and Cell Biology, University of Tartu, Tartu, 51010 Estonia; 4 Institute of Clinical Medicine, Internal Medicine, University of Eastern Finland, Kuopio, Finland; 5 Kuopio University Hospital, Kuopio, Finland; 6 Computational Medicine, Faculty of Medicine, University of Oulu and Biocenter Oulu, Oulu, Finland; 7 NMR metabolomics Laboratory, School of Pharmacy, University of Eastern Finland, Kuopio, Finland; 8 Department of Cellular and Molecular Medicine, Cleveland Clinic, Cleveland, OH 44195 USA; 9 Computational Medicine, School of Social and Community Medicine, University of Bristol and Medical Research Council Integrative Epidemiology Unit at the University of Bristol, Bristol, UK; 10 Department of Human Genetics, University of California, Los Angeles, Los Angeles, CA 90095 USA; 11 Department of Microbiology, Immunology and Molecular Genetics, University of California, Los Angeles, Los Angeles, CA 90095 USA

**Keywords:** Host-microbiota interactions, TMAO, Metabolic traits, Serum metabolites, Type 2 diabetes

## Abstract

**Background:**

The gut microbiome is a complex and metabolically active community that directly influences host phenotypes. In this study, we profile gut microbiota using 16S rRNA gene sequencing in 531 well-phenotyped Finnish men from the Metabolic Syndrome In Men (METSIM) study.

**Results:**

We investigate gut microbiota relationships with a variety of factors that have an impact on the development of metabolic and cardiovascular traits. We identify novel associations between gut microbiota and fasting serum levels of a number of metabolites, including fatty acids, amino acids, lipids, and glucose. In particular, we detect associations with fasting plasma trimethylamine N-oxide (TMAO) levels, a gut microbiota-dependent metabolite associated with coronary artery disease and stroke. We further investigate the gut microbiota composition and microbiota–metabolite relationships in subjects with different body mass index and individuals with normal or altered oral glucose tolerance. Finally, we perform microbiota co-occurrence network analysis, which shows that certain metabolites strongly correlate with microbial community structure and that some of these correlations are specific for the pre-diabetic state.

**Conclusions:**

Our study identifies novel relationships between the composition of the gut microbiota and circulating metabolites and provides a resource for future studies to understand host–gut microbiota relationships.

**Electronic supplementary material:**

The online version of this article (doi:10.1186/s13059-017-1194-2) contains supplementary material, which is available to authorized users.

## Background

Multiple studies in humans and animal models have established that the gut microbiota contribute significantly to a variety of cardio-metabolic traits, including obesity, type 2 diabetes (T2D) [1–3], insulin resistance [4–7], atherosclerosis, and heart failure [8–10]. A growing body of evidence shows that microbial metabolites have a major influence on host physiology. The best known bacterial fermentation products are short chain fatty acids (SCFAs) such as acetate, butyrate, and propionate, which exert several effects, including maintenance of gut barrier function and providing a source of energy for colonocytes and bacterial communities (reviewed in [11]). Another example is trimethylamine-N-oxide (TMAO), a metabolite derived from dietary choline and carnitine through the action of gut microbes. TMAO plays an important role in several cardio-metabolic phenotypes and is associated with chronic kidney disease [8, 9, 12, 13].

The majority of published studies thus far have focused on describing the gut microbiome profile changes between specific disease groups and control individuals. These findings usually explain dysbiosis states related to end phenotypes and are not well powered to discover alterations that are present in the general population and can ultimately affect the development of common complex diseases. A growing body of evidence suggests that a variety of intrinsic and environmental factors, such as long-term dietary patterns and host physiology and genetics, significantly affect the structure and functional capabilities of gut microbial communities [14–17].

Recently, two large-scale studies from Europe characterized the gut microbiota composition and variation in subjects collected from the general population [18, 19]. Rich metadata on various health and lifestyle factors showed several associations that impact the variation of the gut microbiome in the general population. The Netherland cohort study also showed the contribution of gut microbiota to body mass index (BMI) and lipid variation, highlighting its role in the regulation of metabolic processes and cardiometabolic diseases [20]. To date, however, no population-based studies have been performed to assess the association between microbiota and a large spectrum of cardiometabolic traits.

In the current study we performed systematic analysis of the gut microbiome and a variety of metabolically relevant traits in 531 middle-aged Finnish men, collected from the general population of the METabolic Syndrome In Men (METSIM) study [21]. All subjects from this population-based cohort have been extensively characterized for a variety of cardiovascular and metabolic traits, including 2-h oral glucose tolerance test (OGTT), insulin resistance, BMI, and serum metabolites such as fatty acids, lipids, amino acids, glycolysis precursor metabolites, and ketone bodies. We identified several significant relationships, particularly between gut microbiota and circulating serum metabolites. In addition, we examined the fasting plasma levels of the microbiota-dependent metabolite TMAO. Several observed relationships were further supported by operational taxonomy unit (OTU)-based co-occurrence network analysis. Finally, we also assessed microbiome–metabolite interactions in subjects with extreme BMI and in a pre-diabetic stage based on an impaired fasting glucose tolerance test.

## Results

### Landscape of the METSIM gut microbiome

Our study included 531 individuals, representing a sub-cohort who participated in a follow-up study from the METSIM cohort (total cohort n = 10,000). This general population-based study cohort, consisting of males aged 45–70 years, was collected from Eastern Finland and has been extensively phenotyped for a variety of metabolic parameters (Additional file 1: Table S1) [21]. Stool samples for microbiota analysis were collected during a clinical visit together with fasting blood samples. Baseline characteristics for each study participant are summarized in Table 1.

**Table 1:** Characteristics of 531 METSIM subjects

Clinical trait	Average	Standard deviation
Age (years)	61.97	5.45
Body mass index (kg/m^2^)	27.92	3.60
Waist to hip ratio (cm)	0.998	0.06
Fat mass (%)	25.79	6.93
OGTT fasting plasma glucose (mmol/l)	5.77	0.49
OGTT 30 min plasma glucose (mmol/l)	9.35	1.49
OGTT 120 min plasma glucose (mmol/l)	5.96	1.91
OGTT fasting plasma insulin (mU/l)	9.43	5.97
OGTT 30 min plasma insulin (mU/l)	65.20	42.79
OGTT 120 min plasma insulin (mU/l)	48.01	66.76
HbA1c (%)	5.6	0.29
Systolic blood pressure (mmHg)	130.12	13.51
Diastolic blood pressure (mmHg)	82.35	7.84
HOMA-IR	2.47	1.69

*Hba1c* glycated hemoglobin, *HOMA-IR* homeostatic model assessment of insulin resistance

We first characterized the phylogenetic variation across samples at different taxonomic levels. We sorted sequences into 1148 OTUs (≥97% identity). Of these OTUs, 321 were present in at least 50% of the samples. As expected, we observed considerable variation in the abundance of taxa in the METSIM fecal microbial communities, indicating a typical Western diversity profile where *Firmicutes* (mean = 53.43%, range = 12.9–94.1%) and *Bacteroidetes* (mean = 40.80%, range = 0.11–85.9%) were the dominant phyla (Additional file 1: Table S2; Additional file 2: Figure S1a). Overall, we detected ten bacterial phyla and one archaeal phylum. Forty percent of individuals contained archaeal taxa from phylum *Euryarcheota* and genus *Methanobrevibacter* (0.15%, 0–6.7%). The most dominant bacterial families (90% of total sequences) belong to *Bacteoridacea* (28% of total sequences), *Ruminococcacea* (20% of total sequences), and *Lachnospiracea* (16% of total sequences) (Additional file 2: Figure S1c). At the genus level, *Bacteroides* was the most dominant and variable phylotype across 531 METSIM samples ranging from 0.1 to 85.6%, in agreement with previous results [22, 23].

**Fig. 1:**
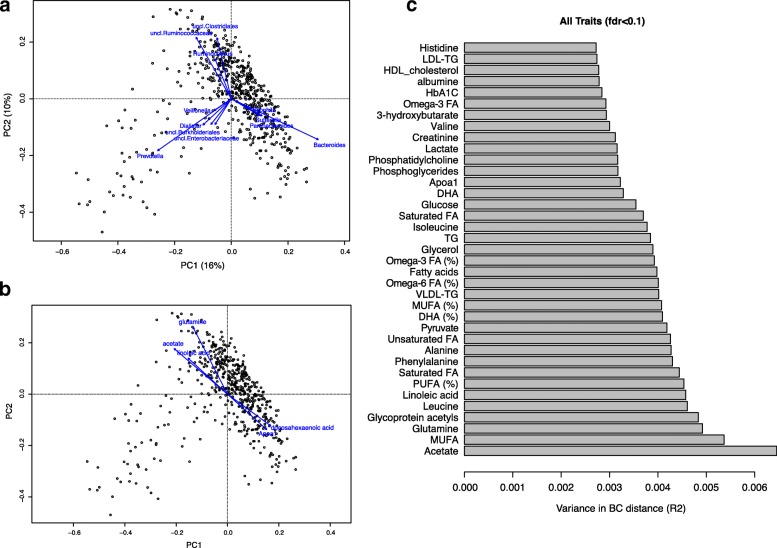
Microbial community variation in the METSIM cohort. **a** Top contributors to community variation as determined by canonical correspondence analysis on unscaled genera abundances, plotted on the first principal component (*PC*) dimensions (*arrows* scaled to contribution). **b** The top seven metabolite contributors to microbiome community variation. **c** A total of 32 out of 60 factors (60%) explained a total of 13% of variance of the gut microbiome according to Bray–Curtis distance. *TG* triglycerides, *FA* fatty acids, *DHA* docosahexaenoic acid, *MUFA* monounsaturated fatty acid, *PUFA* polyunsaturated fatty acid

We first assessed how variable the gut microbial composition was in the METSIM cohort in terms of microbial diversity and richness. The microbial richness, which refers to the number of OTUs per individual, exhibited on average 329 OTUs per individual, ranging from 108 to 474 (Additional file 1: Table S3). Based on unconstrained canonical analysis of genus-level community composition (see “Methods”), we found that the main genera driving diversity in the gut landscape are *Bacteroides*, an unclassified *Ruminococcaceae* genus, and *Prevotella* (Fig. 1a). This is consistent with other population-based gut microbiome studies, showing that these three genera are major contributors to community variation and define previously proposed enterotypes [18, 23]. However, our data support continuous rather than distinct clusters, in agreement with recently published data [24].

### Metabolite associations with gut microbiota richness and diversity

The METSIM cohort has been studied for a variety of cardiovascular and metabolic traits, providing an opportunity to identify potential relationships with gut microbiota composition. Using a nuclear magnetic resonance (NMR) spectroscopy platform [25], we quantified a broad molecular signature of the systemic serum metabolite profiles, such as lipids, fatty acids, glycolysis-related metabolites, ketone bodies, and amino acids. A list of the traits we examined is presented in Additional file 1: Table S1.

We first investigated whether bacterial diversity and richness were correlated with 57 traits (Additional file 1: Table S1). After adjustment for age and treatment, 37 traits were associated with inter-individual distance of microbial composition (Bray-Curtis distance) at a false discovery rate (FDR) of 0.1, together explaining 14.1% of the variation in composition distance (Additional file 1: Table S4). Figure 1b shows the traits that contribute the highest variation in microbial composition distance. Among the associations we detected, acetate showed the strongest effect on the variation in microbial diversity and the richness was positively correlated with glutamine, glycated hemoglobin (Hba1c), and acetate levels (FDR <0.1) (Fig. 1b, c; Additional file 1: Tables S4 and S5; Additional file 2: Figure S2).

### Metabolite associations with gut microbiota composition

**Fig. 2:**
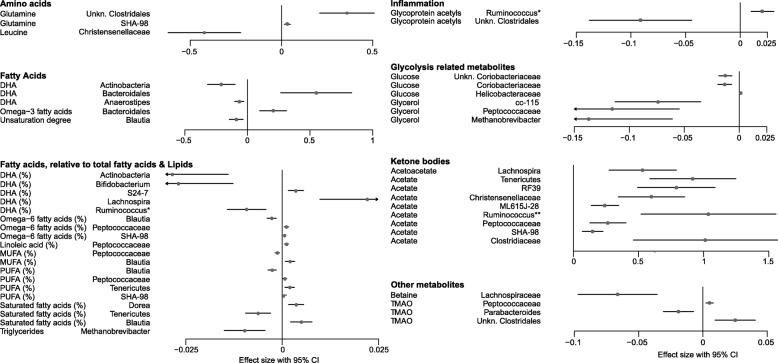
Associations between microbiota taxa and circulating serum metabolites. Serum metabolite concentrations were measured from fasting samples using NMR or LC-MS/MS. They were then associated with microbial taxa following adjustment for age and treatment of the subjects. *Ruminococcus from the family *Lachnospiraceae*; **Ruminococcus from the family *Ruminococcaceae. CI* confidence interval, *DHA* docosahexaenoic acid, *MUFA* monounsaturated fatty acid, *PUFA* polyunsaturated fatty acid

We next performed multivariate association analysis between each trait with 91 unique taxa and 321 OTUs (shared in 50% of individuals). When adjusted for age and treatment, we identified 140 associations between 26 traits and 72 unique bacterial OTUs at a FDR of 0.05 (Additional file 1: Table S6). Overall, 51 OTUs were significantly associated with different fatty acids, 33 OTUs with ketone bodies, 19 OTUs with amino acids and glycolysis-related metabolites, nine OTUs with glycoprotein acetyls, six OTUs with choline pathway metabolites, and three OTUs with HbA1c levels (Additional file 1: Table S6; Additional file 2: Figure S3). At the taxonomy level, we identified a total of 40 significant associations between 17 traits and 23 unique taxa with FDR <0.05: 19 taxa were associated with fatty acids, nine with ketone bodies, five with glycolysis-related metabolites, three with amino acids, two with glycoprotein acetyls, and one each with betaine and TMAO (Fig. 2; Additional file 1: Table S7).

The strongest associations with both taxonomy and unique bacterial OTUs were with acetate and glutamine levels, indicating a strong overlap between associations detected with diversity and richness. Acetate is the most common SCFA in the human colon, produced in the large intestine by anaerobic intestinal microbiota through fermentation of non-digestible carbohydrate. In total, 28 unique OTUs and eight unique taxa were associated with serum acetate levels with FDR <0.05 (Fig. 2; Additional file 1: Tables S6 and S7). Increased acetate levels were associated with higher abundances of phylum *Tenericutes* (mainly represented by order RF-39), family *Christensenellaceae*, unclassified *Clostridales*, *Peptococcaceae*, and several members of family *Clostridiaceae*, whereas reduced levels were associated with higher abundances of *Blautia* and genus *Oscillospira* (Additional file 1: Table S6; Additional file 2: Figure S3). Acetate was also strongly correlated with other traits. For example, a significant positive correlation was detected with polyunsaturated fatty acids, such as omega-6 and 18:2 linoleic acids and with serum cholesterol and TMAO levels, whereas a negative correlation was detected with several amino acids (alanine, tyrosine, isoleucine, and leucine), glycolysis precursors (lactate and pyruvate), and glycoprotein acetyl levels (FDR <0.05) (Additional file 1: Table S8).

Western diets tend to have a high fat content, and increased fat consumption is associated with obesity, insulin resistance, and metabolic syndrome [26]. It has been shown that high-fat content food perturbs gut microbiota and induces pro-inflammatory signaling [3, 27]. We identified several significant associations with various fatty acids, accounting altogether for 41% of all taxonomy level (19 out of 46) and 33.8% of all OTU level (51 out of 151) associations (Fig. 2; Additional file 1: Tables S5 and S6; Additional file 2: Figure S3). At both the taxa and OTU levels, the most significant associations were observed with the abundance of members of the genus *Blautia* and phylum *Tenericutes*. The abundance of *Blautia* was positively associated with saturated and monounsaturated fatty acids and negatively associated with degree of unsaturation and polyunsaturated fatty acids, including omega-3, 22:6 docosahexaenoic acid, omega-6, and 18:22 linoleic acids. In contrast, opposite associations with the same traits were detected with phylum *Tenericutes*, family *Peptococcaceae*, and genus *Bacteroides* (Fig. 2).

Multiple significant associations were detected with glycolysis-related metabolites and with amino acids (Fig. 2). For example, fasting glucose levels were strongly associated with unclassified *Coriobacteriaceae* and several OTUs from *Blautia* were positively associated with pyruvate and glycerol. This is in line with a recent finding showing that members of *Coriobacteriacea* are depleted in individuals with diabetes and impaired glucose tolerance [28]. One of the strongest associations between amino acids and gut microbiota was detected with glutamine levels, where several species from unclassified *Clostridales* were positively correlated with increased glutamine concentrations (Fig. 2; Additional file 2: Figure S3). In addition, the branch-chain amino acids (BCAAs) isoleucine and valine were negatively associated with the abundance of *Christensenellaceae* and positively associated with *Blautia*. Recent studies have shown that glutamine supplementation alters gut microbiota composition and improves glucose tolerance and obesity [29, 30]. In contrast, BCAAs and other hydrophobic amino acids, including alanine and the aromatic amino acids phenylalanine and tyrosine, have been shown to be elevated in individuals with metabolic disorders. Our data support these observations showing significantly elevated glutamine and reduced BCAAs in individuals with low BMI and elevated BCAAs in individuals with high homeostatic model assessment of insulin resistance (HOMA-IR) values (Additional file 2: Figure S4).

Recently, population-based cohort studies from the Netherlands investigated the contribution of gut microbiota to the variation of blood lipids and BMI [19, 20]. Here we confirmed associations with triglyceride (TG) levels, showing that higher abundances of genus *Methanobrevibacter* from *Archaea* (*P* = 4.4 × 10^−4^), *Tenericutes* (*P* = 0.0022), *Peptococcaceae* (*P* = 0.0098), and *Christensenellaceae* (*P* = 0.0123) correlate with lower TG levels. Although some other associations with high-density lipoprotein (HDL), low-density lipoprotein (LDL), and BMI were shared between our study and the Netherlands cohort study, after correction for multiple comparisons these associations were no longer significant (FDR >0.1; data not shown).

### Associations with TMAO, a metabolite derived from gut microbiota

Recent studies have revealed associations of gut microbiota-dependent metabolite TMAO with the development of cardiometabolic and renal phenotypes [8, 10, 31–34]. Using mass spectrometry analysis [9], we measured choline, carnitine, betaine, and TMAO levels in fasting serum and identified four significant associations (FDR <0.05) with unique taxa (three with TMAO and one with betaine) and eight associations with OTUs (six with betaine, one with choline, and one with carnitine) (Fig. 2; Additional file 1: Tables S5 and S6; Additional file 2: Figure S3). We confirmed previously detected associations between TMAO and the abundance of *Peptococcaceae* (*P* = 4.67 × 10^−4^) and *Prevotella* (*P* = 0.004) (Fig. 2) [12, 35]. Interestingly, we also observed a negative association between TMAO and the abundance of common gut commensal *Faecalibacterium prausnitzii* (*P* = 0.006; FDR <0.08). Decreased abundance of *F. prausnitzii* has been linked to dysbiosis in several human disorders, including obesity, diabetes, and several immune-related diseases [36, 37]. Our data also confirmed a previously reported association between TMAO and creatinine levels, but in addition we found significant associations with valine, acetate, and unsaturated and omega-3 fatty acids levels (Additional file 1: Table S9; Additional file 2: Figure S5).

### OTU co-occurrence network and metabolite associations

**Fig. 3:**
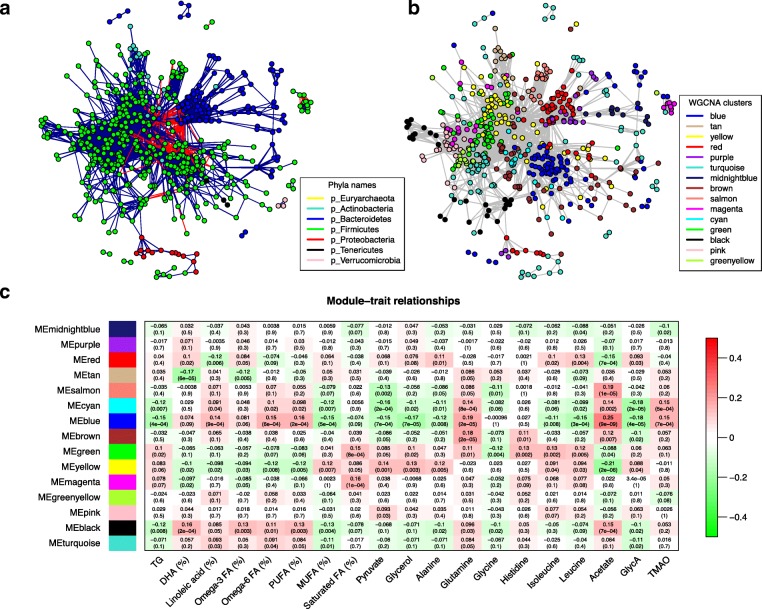
OTU co-occurrence network and module-trait associations. **a** OTU co-occurrence network where OTU (*nodes*) are colored according to the phyla to which they belong. *Blue edges* correspond to positive correlations and *red edges* to negative correlations. Any resulting correlations with *p* value ≥0.01 and abs(r) <0.3 were removed. **b** OTU co-occurrence network were OTU (*nodes*) are colored according to WGCNA module colors. **c** Module–trait associations are shown. Each cell of the matrix contains the correlation between one OTU module and a metabolic trait, and the corresponding *p* value. The table is color-coded by correlation according to the color legend (*red* for positive correlations and *green* for negative correlations). *FA* fatty acids, *DHA* docosahexaenoic acid, *MUFA* monounsaturated fatty acid, *PUFA* polyunsaturated fatty acid, *GlycA* glycoprotein acetyls, *TMAO* trimethyl N-oxide

The human gut is a complex and dynamic ecosystem consisting of a diverse collection of microorganisms with a variety of functional relationships. In order to detect relationships between different members of the gut microbiome and their potential combined effect on metabolic output, we constructed a network of co-occurrence OTUs and interrogated the network for modules using weighted gene co-expression network analysis (WGCNA) (see “Methods”; Fig. 3a, b). The complete list of OTUs and their module organization is available in Additional file 1: Tables S10 and S11. Phylogenetically related OTUs were observed to cluster into the same modules preferentially, although each module also included phylogenetically distinct OTUs from different taxa. Therefore, in addition to phylogenetic relatedness, the formation of modules depended upon additional ecological affinities, reflecting complementary convergent functionality.

The module–metabolite associations confirmed previously detected metabolite–taxa relationships. For example, the blue module, which contains OTUs from unclassified *Clostridales*, *Tenericutes*, *Methanobrevibacter*, and *Christensenellaceae*, is positively correlated with acetate, glutamine, and polyunsaturated fatty acids, whereas the yellow module, representing OTUs mostly from *Blautia*, is negatively correlated with acetate and other traits associated with the blue module (Fig. 3c). All these associations were detected with single taxonomy and/or OTUs (Additional file 1: Tables S5 and S6). However, both modules also included several other OTUs from different taxa, providing further insights for metabolically important relationships.

### The gut microbiota relationships with obese and impaired glucose tolerance phenotypes

We next assessed the associations of gut microbiomes with obese and pre-diabetic states by subdividing individuals based on BMI and the entire range of fasting and 2-h plasma glucose tolerance tests. The average BMI of the 531 subjects was 27.92 (±0.16), where 100 subjects (19%) had a BMI <25 and 132 (25%) had a BMI >30. The pre-diabetic state includes individuals with impaired fasting glucose (IFG), impaired glucose tolerance (IGT), or both (see “Methods” for details) [38]. Based on these criteria, 164 subjects (31%) had normal glucose tolerance (NGT) and 352 had been classified as in a pre-diabetic stage (pre-T2D), from which 287 (54%) had IFG, 15 (2.8%) had isolated IGT, and 50 (9.4%) had a combination of IFG and IGT. A total of 15 individuals had newly diagnosed T2D in follow-up visits and these individuals were excluded from the analysis. The characteristics of these subjects are shown in Additional file 1: Table S12.

**Fig. 4:**
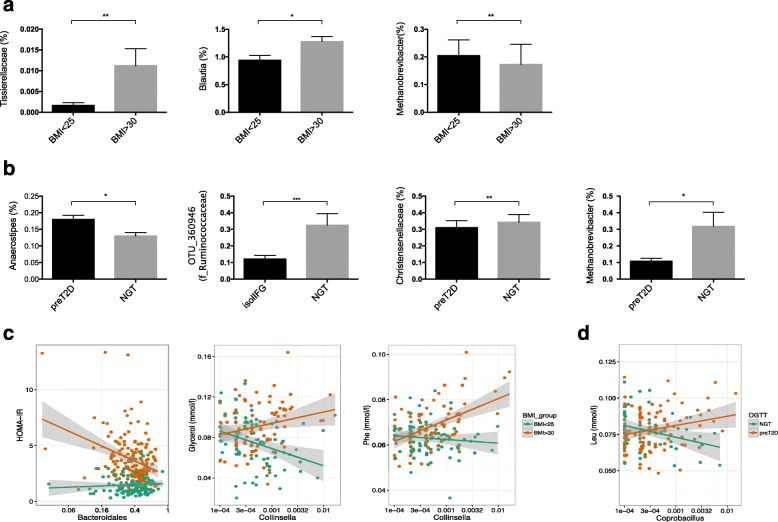
The gut microbiota differences in metabolic phenotypes. Mean proportions of significantly different taxa between individuals with different BMI values (**a**) and individuals with different 2-h glucose tolerance test (NGT and pre-T2D) (**b**). Examples of BMI/OGGT metabolite–microbiota interaction, where the abundances of *Bacteroidales*, *Collinsella* (**c**) and *Coprobacillus* (**d**) exhibit opposite associations in individuals with low versus high BMI levels or individuals with impaired glucose tolerance test (**c**). *P ≤ 0.01, **P ≤ 0.001, ***P ≤ 0.0001. *BMI* body mass index, *GT* glucose tolerance, *NGT* normal glucose tolerance, *T2D* type 2 diabetes

We first evaluated bacterial richness, diversity, and differences in the ratio of *Firmicutes* to *Bacteroidetes* in subjects with different body weights and predisposition to T2D based on OGTT. No significant differences were detected in either bacterial richness or in the ratio of *Firmicutes* to *Bacteroidetes* (Additional file 2: Figure S6a, b). However, several differences were observed in gut microbiota composition between subjects with extreme BMI and between NGT and pre-T2D (Fig. 4a, b; Additional file 2: Figure S6c). For example, subjects with high BMI had significantly higher abundance of family *Tissierellacea* and genus *Blautia* and decreased abundances of Archaea (*Methanobrevibacter*) (Fig. 4a). Pre-diabetic subjects, however, had higher abundances of *Anaerostipes* and lower abundances of an OTU from families *Ruminococcaceae* and *Christencenellacea* and genus *Methanobrevibacter* (Fig. 4b). Both the methanogen *Methanobrevibacter* and *Christencenellacea* have been associated with a lean phenotype in previous studies [6, 16, 19, 39].

Using a multivariate regression model we tested whether obesity and pre-T2D affect associations between individual microbial taxa and metabolites, and whether there was interaction between obese/pre-T2D status and microbial taxa. For example, in obese subjects, the higher abundance of *Bacteroidales* was associated with lower HOMA-IR and the higher abundance of *Collinsella* with higher levels of glycerol and phenylalanine, and in lean subjects the effect was opposite (Fig. 4c). Also, the abundance of *Coprobacillus* relative to plasma leucine concentrations depended on the pre-T2D status (FDR <0.05) (Fig. 4d). Finally, we also constructed OTU co-occurrence networks separately for subjects with NGT and for pre-T2D as defined by OGTTs. Our data show clear differences between interactions of OTUs in both networks (Additional file 1: Table S13; Additional file 2: Figure S7a). A module preservation analysis indicated modules that are unique only for pre-T2D individuals. For example, a tan module, which contains mostly OTUs from family *Ruminococcacea* and genera *Ruminococcus* and *Oscillospira*, is strongly correlated with glucose levels only in pre-T2D subjects (Additional file 2: Figure S7b–d).

## Discussion

We investigated the associations of gut microbiota with a large number of metabolically relevant traits using a subset of 531 Finnish men the from Metabolic Syndrome in Men (METSIM) cohort. The METSIM cohort (total 10,197 individuals) is one of the largest single-site population based cohorts and was collected from Kuopio, Eastern Finland, and established to evaluate genetic determinants of metabolic traits in middle-aged men. There is increasing evidence that gut microbiota are an important factor, in addition to genetic and life style factors, for the development of obesity, insulin resistance, T2D, and cardiovascular disease. The gut microbiome is a complex and metabolically active community, producing many metabolites which can directly influence host phenotype. Our study investigated gut microbiota relationships with a variety of factors that have profound impacts on the development of metabolic and cardiovascular traits. We present several new findings. First, we identified a number of associations between gut microbiota and fasting serum levels of fatty acids, amino acids, lipids, and glucose. These associations were detected with both the diversity and richness of gut microbiota as well as with unique bacteria. Second, we detected significant associations with fasting plasma TMAO concentrations, a metabolite derived from dietary choline and carnitine through the action of gut microbiota. Third, we identified a group of co-occurrence microbes and demonstrated that OTU-based module–trait associations confirm already identified associations as well as provide some new insights for microbiota–trait relationships. Finally, we detected altered microbiota composition and significant microbiota–metabolite relationships dependent on BMI and normal or altered oral glucose tolerance. These points are discussed in turn below.

Mounting evidence in mice and humans shows the important role of gut microbiota-derived metabolites in regulating metabolism. Multiple studies have shown that plasma levels of TMAO, derived from dietary choline and carnitine through the action of gut microbiota, are associated with coronary artery disease, stroke, and several cardiometabolic traits, including increased coagulation and vascular inflammation [8, 31–33]. Consistent with previous findings in both mice and humans, we found that the plasma TMAO concentrations were significantly associated with *Prevotella* and *Peptococcaceae*. Additionally, we saw a significant positive correlation between plasma TMAO concentrations with unclassified *Clostridales* and negative correlation with *F. prausnitzii* (FDR <0.1). *F. prausnitzii* has been shown to exert anti-inflammatory effects and decreased abundance of it has been noted in several immune-related diseases.

Our data show the strongest associations between gut microbiota and acetate and glutamine levels, where a total of nine unique taxa were significantly associated with elevated acetate levels. The higher glutamine and acetate levels were both also associated with significantly higher bacterial richness. An elevated abundance of unclassified *Clostridales* was associated with elevated glutamine levels and negatively associated with inflammation-related circulating levels of glycoprotein acetyls (mainly alpha-1-acid glycoprotein). The phylum *Tenericutes* (mainly represented by order *RF-39* and *ML615J_28*) and family *Christensenellaceae* were among the top taxa that showed association with elevated acetate levels. Both taxa have been previously reported to be associated with low BMI and TG and higher HDL levels [16, 20]. In the current study we were only able to confirm order *ML615J_28* associations with lower TG levels (*P* = 0.0093), although other trends were consistent. In addition, we identified novel strong associations between *Christensenellaceae* and lower levels of BCAAs (leucine and isoleucine) and between *Tenericutes* and higher ratios of polyunsaturated fatty acids to total fatty acids.

Acetate is the most common short-chain fatty acid (SCFA) that is formed during bacterial fermentation of carbohydrates in the colon [40]. SCFAs are readily absorbed in the plasma of the host via the intestinal epithelium and thus can serve as an energy source, predominantly via metabolism in the liver [11, 41]. Previous studies have shown that both increases and decreases in plasma SCFA concentrations can be associated with obesity and metabolic syndrome [42]. It has been shown that prebiotic fructooligosaccharides and inulin increase the levels of acetate and that this is associated with reduced body weight and fat mass, decreased diabetes, and a lower food intake [43, 44]. However, recent work by Perry et al. [45] showed that increased production of acetate by an altered gut microbiota in rodents leads to activation of the parasympathetic nervous system and stimulation of insulin secretion. These studies indicate that the role of acetate in driving obesity depends on the gut microbiota composition and on dietary fiber intake.

Several studies have indicated influences of bacterial taxa on lipid and fatty acid levels [20, 46]. The serum fatty acid profile is determined by both diet and host factors, such as endogenous fatty acid metabolism [47]. The Western diet, rich in simple carbohydrates and fat, markedly affects the gut microbiota ecosystem at the compositional and functional levels [3, 14, 48, 49]. Elevated levels of lipids and free fatty acids are known risk factors for metabolic syndrome, insulin resistance, and obesity [50]. However, dietary fatty acids differ in structure and have been shown to exert opposite metabolic effects [51, 52]. Our data show that fasting serum levels of glycerol, monounsaturated fatty acids, and saturated fatty acids are strongly associated with increased abundance of *Blautia* and *Dorea* and decreased abundance of *Coprococcus* and *Peptococcaceae*, whereas the same taxa show opposite associations with polyunsaturated fatty acids, including omega-6 and DHA and omega-3 and linoleic acid. Several OTUs from genus *Blautia* were also positively associated with BCAA (isoleucine and leucine), alanine, glycerol, and pyruvate levels. *Blautia* is a common gut habitant, an acetogen that belongs to the family *Lachnospiraceae*, which is one of the major taxonomic groups of the human gut microbiota that degrade complex polysaccharides to SCFAs. *Blautia* has the ability to ferment a large variety of organic substrates, enabling flexible growth in the colon. Bacteria in the *Blautia* genus have been associated with both decreased and increased obesity and Crohn’s disease [53, 54]. Our data also showed increased abundance of *Blautia* in individuals with high BMI levels (Fig. 4a). Interestingly, a recent study detected an association between *Blautia* and human genetic variants in a genomic region that has been associated with obesity and BMI [55]. Since there is great diversity among *Blautia* oligotypes, suggesting that the genus represents strains that comprise a variety of metabolic capacities optimized for a host and a host environment [56].

Complex interactions exist between gut microbiota and metabolites. Transient changes in the intestinal ecosystem occur throughout life and are affected by several factors, the most by dietary components. Consumption of fat- and sugar-rich diets modifies the gut microbiota community, which triggers changes in host metabolic pathways and ultimately affects metabolic state. TMAO is a good example, where multiple bacterial enzymes are linked to specific biochemical transformations by the host. However, the exact molecular relationship among microbe-derived gut metabolites, how microbes affect host signaling pathways, and host physiology are still poorly understood. Our data are correlative and therefore we are unable to determine the functional potential of the microbial community. Further studies (metagenomic and functional studies) will be required in order to understand the exact link between microbiota, metabolites, and their link with host health.

Bacteria in the gut constitute a complex ecosystem in which different species exhibit specialized functions and interact as a community. Therefore, genetic studies of communities, as defined by co-occurrence or some other measures, may provide a better global picture of the functional variation that occurs in populations [57]. We identified modules of microbial communities based on co-occurrence and used these groups to identify associations with traits. These generally confirmed similar associations observed with single taxa or OTUs; for instance, OTUs that belong to blue and yellow modules have opposite effects with traits and these modules contain taxa that showed significant associations with the same traits (Additional file 1: Table S11). In addition, module-based associations also pointed to some new associations that were missed with single taxa or OTU-based associations.

The METSIM study has been established to evaluate important determinants of metabolic and cardiovascular traits. T2D is preceded by a long pre-diabetic state characterized by mild elevation of fasting and/or postprandial glucose levels. Since this asymptomatic stage may last for years, it’s important to determine characteristics that associate with impaired glucose metabolism. In this study the risk of T2D was assessed using OGTTs and 54% of subjects had either impaired fasting glucose (IFG) and/or impaired glucose tolerance (IGT). We were unable to detect significant differences in bacterial richness as well as changes in the ratio of *Firmicutes* to *Bacteroidetes*, parameters that are usually linked to obesity and T2D phenotypes. The 16S rRNA gene captures broad shifts in community diversity over time, but with limited resolution and lower sensitivity compared to metagenomic data [58]. Therefore, metagenomic approaches provide better estimates of the extent of microbial diversity and richness. However, several taxa showed significantly different abundances between lean and obese subjects and individuals with impaired glucose tolerance. Moreover, we also showed specific gut microbiota–metabolite interactions within these groups.

Finally, we were able to confirm some previously detected associations with plasma lipids. For example, the increased abundance of *Methanobacteriaceae* and genus *Coprococcus* was associated with lower levels of TGs. In addition, novel significant associations were detected between *Methanobacteriaceae* and lower levels of glycerol and total and monounsaturated fatty acid levels (FDR <0.1). In our cohort 40% of subjects had significant levels of *Methanobacteriaceae*, a common methanogen which recycles hydrogen by combining it with carbon dioxide to form methane. *Methanobacteriaceae* have been associated with reduced levels of obesity [59]. Consistent with our associations with TG levels, inhibitory effects of saturated fatty acids on methanogenesis have been recently reported [60]. However, we were unable to detect any significant associations with HDL and total cholesterol levels. The observed inconsistencies may indicate either limited power to detect relationships, variations between study designs, and/or the existence of population-specific associations. The METSIM cohort involves only middle-aged Finnish men, whereas previously reported population-based studies from Europe report findings for both genders over a larger age distribution. The inconsistencies are also commonly seen in animal studies, where mice are maintained in highly controlled environments with similar diets. In the current study, all stool samples were collected in the clinic at the same time as all measurements and blood were taken from the Kuopio region of Eastern Finland. This population is a genetically relatively homogeneous sub-isolate, which originated from a limited number of founder ancestors and is, therefore, enriched in specific genetic variants. Additional population-based cohort studies are needed to determine whether there are in fact substantial differences in predictors of fecal sample-derived microbiota between populations. Importantly, our study involved only middle-aged Finnish men and therefore we cannot exclude the possibility that some of the detected associations are gender-specific. Recent evidence clearly indicates gender differences in bacterial richness, diversity, and composition [19, 61–63].

## Conclusions

Our study provides a strong indication that several cardio-metabolically relevant traits and metabolites are modulated by the action of gut microbiota. We have identified a number of novel relationships between gut microbes and different circulating metabolites, where some of these interactions could predict a pre-diabetic state. Our data provide a significant biological resource and novel avenues for further studies. Further functional and mechanistic investigations are needed in order to clarify relationships between microbial communities and specific cardio-metabolic phenotypes. The extensive clinical and molecular characterization of the METSIM cohort, as well as its homogenous nature, make it particularly useful for understanding host–microbiota relationships.

## Methods

### Sample collection and characterization

A total of 531 men from the ongoing population-based cross-sectional METSIM study were included in the current study. METSIM is a randomly selected cohort of unrelated men (aged 45–70 years) selected from the population register of the town of Kuopio in Eastern Finland (population 95,000). Our subset of participants took part in a 7-year follow-up study. The study was designed to determine the prevalence and genetic determinants of a wide spectrum of metabolic and cardiovascular diseases (metabolic syndrome, T2D, impaired glucose tolerance, impaired fasting glucose, hypertension, obesity, dyslipidemia, coronary heart disease, stroke, and peripheral vascular disease). Every participant had a 1-day outpatient visit to the Clinical Research Unit at the University of Kuopio, including an interview about the history of previous diseases, current health status, and drug treatment. Each participants’ height, weight, waist to hip circumference, and blood pressure were measured together with an evaluation of glucose tolerance and cardiovascular risk factors. The study protocol has been previously described (Stancáková et al. [21]). Characteristics of the subjects included in this study are shown in Table 1. Fasting blood samples were drawn after 12 h of fasting followed by an oral glucose tolerance test (OGTT). Stool samples were provided during their evaluation at University of Kuopio Hospital and immediately stored at −80 °C. All subjects have given written informed consent and the study was approved by the Ethics Committee of the University of Kuopio and was in accordance with the Helsinki Declaration.

### Sample preparation, sequencing, and data processing

Microbial DNA was extracted from a total of 531 frozen fecal samples using the PowerSoil DNA Isolation Kit (MO BIO Laboratories, Carlsbad, CA, USA) following the manufacturer’s instructions. Microbial DNA was extracted and the 16S rRNA gene was amplified using the 515 F/806R primer set targeting the V4 hypervariable region and the DNA sequenced using the Illumina MiSeq platform as previously described [15]. 16S rRNA sequencing data for the 531 samples are available in the Sequence Read Archive (SRA) under accession number SRP097785 (https://www.ncbi.nlm.nih.gov/sra/?term=SRP097785).

De-multiplexing 16S rRNA gene sequences, quality control, and OTU binning were performed using the open source pipeline Quantitative Insights Into Microbial Ecology (QIIME) version 1.7.0 [64, 65]. The total number of sequencing reads was 12,785,442 (an average of 21,543 reads per sample) with an average length of 153 base pairs. Sequences were binned into OTUs based on 97% identity using UCLUST [66] against the Greengenes reference database (version 13.8) [67]. Each sample’s sequences were rarefied to 10,000 reads per strain to reduce the effect of sequencing depth. Microbial composition at each taxonomic level was defined using the summarize_taxa function in QIIME. To ensure comprehensive analysis, we sequenced biological replicates on 13 samples and observed high reproducibility (Additional file 2: Figure S1b).

### Metabolite measurements

Fasting serum samples were collected in the clinic and stored at −80 °C. These were thawed overnight in a refrigerator prior to analysis for lipids, lipoproteins, fatty acids, amino acids, and glycolysis precursor molecules listed in Additional file 1: Table S1 using a NMR spectroscopy platform [25, 68, 69]. Stable isotope dilution liquid chromatography with on-line tandem mass spectrometry (LC-MS/MS) was used for quantification of plasma TMAO, choline, betaine, and carnitine as previously described [9].

### Glucose metabolism assessment

Plasma glucose was measured by enzymatic hexokinase photometric assay (Konelab Systems reagents; Thermo Fisher Scientific, Vantaa, Finland). HbA1c was analyzed with a Tosoh G7 glycohemoglobin analyzer (Tosoh Bioscience, San Francisco, CA, USA). Plasma insulin concentrations were measured by a luminometric immunoassay measurement (ADVIA Centaur Insulin IRI, no. 02230141; Siemens Medical Solutions Diagnostics, Tarrytown, NY, USA). HOMA-IR calculation was performed according to the formula: Concentration of fasting blood glucose (mmol/l) × Concentration of fasting blood insulin (mU/l)/22.5. Insulin resistance (IR) was diagnosed if HOMA-IR >2.5.

A 75-g OGTT was performed with blood glucose measurement before glucose intake and 2 h later. Patients were divided into three groups depending on the glucose metabolism deviation degree: i) individuals without glucose intolerance (fasting glucose <5.6 mmol/l and 2-h glucose is <7.8 mmol/l); ii) individuals with pre-diabetes (pre-T2D), impaired fasting glucose and/or impaired glucose tolerance (5.6 to 6.9 mmol/l and 2-h glucose from 7.8 to 11.0 mmol/l); and iii) individuals with newly diagnosed T2D at the clinical visit (fasting glucose ≥7.0 mmol/l or 2-h glucose ≥11.1 mmol/l). Individuals with T1D were excluded from the study.

### Statistical analysis

Alpha-diversity (the number of observed genera (richness), Pielou’s index (evenness), and Fisher’s alpha (diversity) and beta-diversity (Bray–Curtis dissimilarities) indices were generated using *vegdist* and *diversity* functions in the *vegan* R package (version 2.3-2). We assessed how many variations of Bray–Curtis distance can be explained by each of the 60 traits using the function *adonis* from the R package *vegan*. The *p* value was determined by 1000× permutations and was further adjusted for multiple testing of 60 traits using the Benjamini and Hochberg method. The total variation explained was also calculated per category (fatty acids, glycolysis-related metabolites, amino acids, ketone bodies) and for all traits together. The association analysis was assessed by the Spearman correlation between each trait and each diversity or richness measure. The *p* values were further adjusted for multiple testing of 60 traits using the Benjamini and Hochberg method.

The contribution of each genus and metabolite to microbiome variation was derived from canonical correspondence analysis (CCA) using both raw as well as normalized genus abundances. CCA was performed using the *vegan* R package function *cca*, which performs ordination based on abundances and variations of each taxa. Covariates of microbiome variation were identified by calculating the association between traits and genus-level community ordination (principal component analysis based on Bray–Curtis dissimilarity) with the *enfit* function in the *vegan* R package (10,000 permutations, FDR 5%). Overall, 60 metadata covariates were identified. Their combined effect size was estimated with the *bioenv* functions in the vegan package. Microbiome covariates were further tested for associations with alpha-diversity measures and abundance of genera.

To test the association for each taxonomy and species, we first filtered low abundance species and did our analysis using 321 OTUs (shared in more than 50% of samples). The percentage of each species was arcsine-square root-transformed by taking the arcsine of the square root of the proportional value of each species. The associations of individual species with each factor were assessed using boosted additive generalized linear models available in the *MaAsLin* R package with 5% significance level after multiple testing correction. *MaAsLin* is a multivariate statistical framework that finds associations between clinical metadata and microbial community abundance or function. In our analysis we used the default settings of MaAsLin.

### Co-occurrence network analysis

Weighted gene co-expression network analysis (WGCNA) [70] was used to generate a co-occurrence network based on the OTU arcsine-square root-transformed count data. Briefly, we considered a signed network and chose the minimal beta value satisfying the scale free topology criteria (optimal beta = 6). We set up deepSplit = 2 and minModuleSize = 10 as parameters for the dynamic tree cut function [71]. The module eigenOTU defined as the first principle component of a module was used to calculate the Pearson correlation between a module and a metabolic trait. Significance of the correlation was determined by a Student asymptotic *p* value. Visualization of the network was performed using the *gplot* function available in the *sna* R package.

For co-occurrence network comparisons, we constructed networks in pre-T2D and NGS conditions using the same criteria as described above. We randomly chose 100 samples in both conditions to ensure the same statistical power in network estimation. Module preservation was assessed using Z-summary as implemented in the WGCNA R package [72]. Briefly, this statistic evaluates the module preservation based on density and connectivity criteria and defined a preservation cutoff by randomly permuting the module labels in the test network (in the present study we used 1000 permutations in the test network, e.g., NGS condition).

## Additional files

Additional file 1: Table S1. List of measured traits. **Table S2.** Relative abundances of detected taxonomy across 531 METSIM subjects. **Table S3.** Relative abundances of detected OTUs across 531 METSIM subjects. **Table S4.** Association of 37 traits with Bray–Curtis distance (FDR <0.1). **Table S5.** Association with OTU richness and Shannon’s diversity index. **Table S6.** The significant associations with individual OTUs (FDR <0.1). **Table S7.** The significant associations with individual taxons (FDR <0.1). **Table S8.** Spearman correlations of acetate with 59 traits. **Table S9.** Spearman correlations of TMAO with 59 traits. **Table S10.** WGCNA modules and the list of OTUs in each module. **Table S11.** Representation of taxonomy in each WGCNA module. **Table S12.** Characteristics of individuals based on the OGTT. **Table S13.** Representation of taxonomy in each WGCNA modules in subjects with NGT and pre-T2D. (XLSX 3213 kb)

Additional file 2: Figure S1. Variability of gut microbiota composition in METSIM samples. **Figure S2.** Association of bacterial diversity and richness measures with traits. **Figure S3.** Associations of OTUs with fasting blood levels of metabolites. **Figure S4.** Glutamine and branched chain amino acid (BCAA) levels. **Figure S5.** Correlation heatmap demonstrating the association between the metabolic traits and fasting TMAO concentrations. **Figure S6.** Gut microbiota profile in obese and T2D. **Figure S7.** Comparison between pre-T2D and NGS OTU networks. (PDF 3209 kb)
