# Supplementary material for: Relationships between gut microbiota, plasma metabolites, and metabolic syndrome traits in the METSIM cohort
Source: Genome Biol. 2017 Apr 13;18:70. doi: 10.1186/s13059-017-1194-2 (PMC5390365; doi:10.1186/s13059-017-1194-2)
Supplement: Supplementary file 2 — Variability of gut microbiota composition in METSIM samples. Figure S2. Association of bacterial diversity and richness measures with traits. Figure S3. Associations of OTUs with fasting blood levels of metabolites. Figure S4. Glutamine and branched chain amino acid (BCAA) levels. Figure S5. Correlation heatmap demonstrating the association between the metabolic traits and fasting TMAO concentrations. Figure S6. Gut microbiota profile in obese and T2D. Figure S7. Comparison between pre-T2D and NGS OTU networks. (PDF 3209 kb) [file 13059_2017_1194_MOESM2_ESM.pdf]

FIGURE S1: Variability of gut microbiota composition in Metsim samples……………………..2

FIGURE S2: Association of bacterial diversity and richness measures with traits……………3

FIGURE S3: Associations of OTUs with fasting blood levels of metabolites…………………… 4

FIGURE S4: Boxplots of glutamine and branched chain amino acid (BCAA) levels …………5

FIGURE S5: Correlation heatmap demonstrating the association between the metabolic traits and fasting TMAO concentrations……………………………………………………………………………………..6

FIGURE S6: Gut microbiota profile in obese and type 2 diabetes…………………………………..7

FIGURE S7: Comparison between preT2D and NGS OTU networks ……………………………….8-9

Figure S1: Variability of gut microbiota composition in Metsim samples. Columns represent the relative abundance of microbial phyla on 538 Finnish males (A) and relative abundance of microbial family on 13 replicated samples (B). The average relative abundance for the top ten most prevalent bacterial family (C).

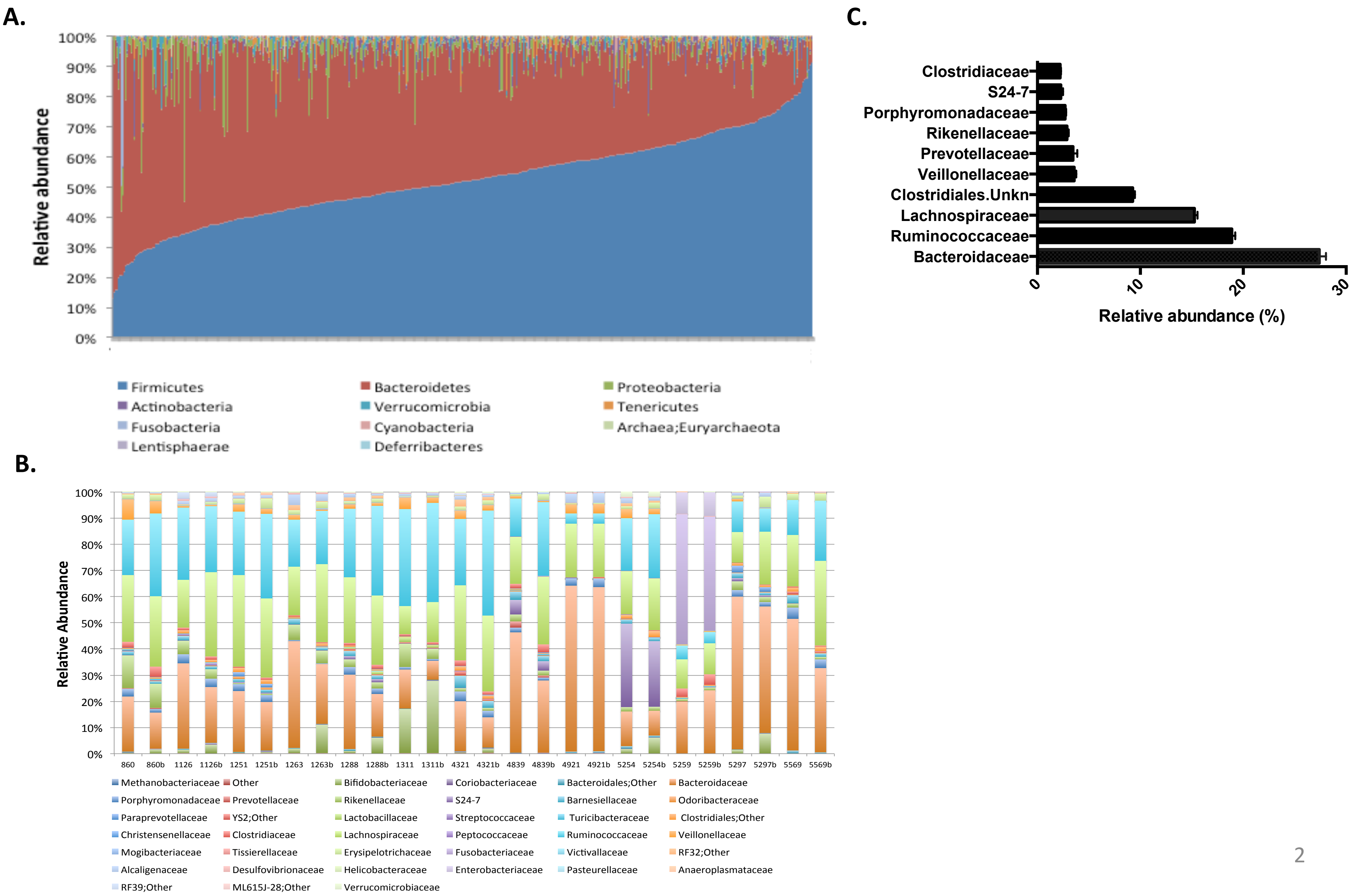

Figure S2: Association of bacterial diversity and richness measures with traits. The heatmap shows the spearman correlation of each trait with alpha, richness (observed number of OTUs) and Shannon' s index of diversity (A). The color key for correlation is shown. Significant differences in bacteria diversity and richness between groups with different HbA1C, glutamine and acetate levels (FDR<0.05)(B). Groups are divided as high or low based on mean metabolite concentrations.

A.

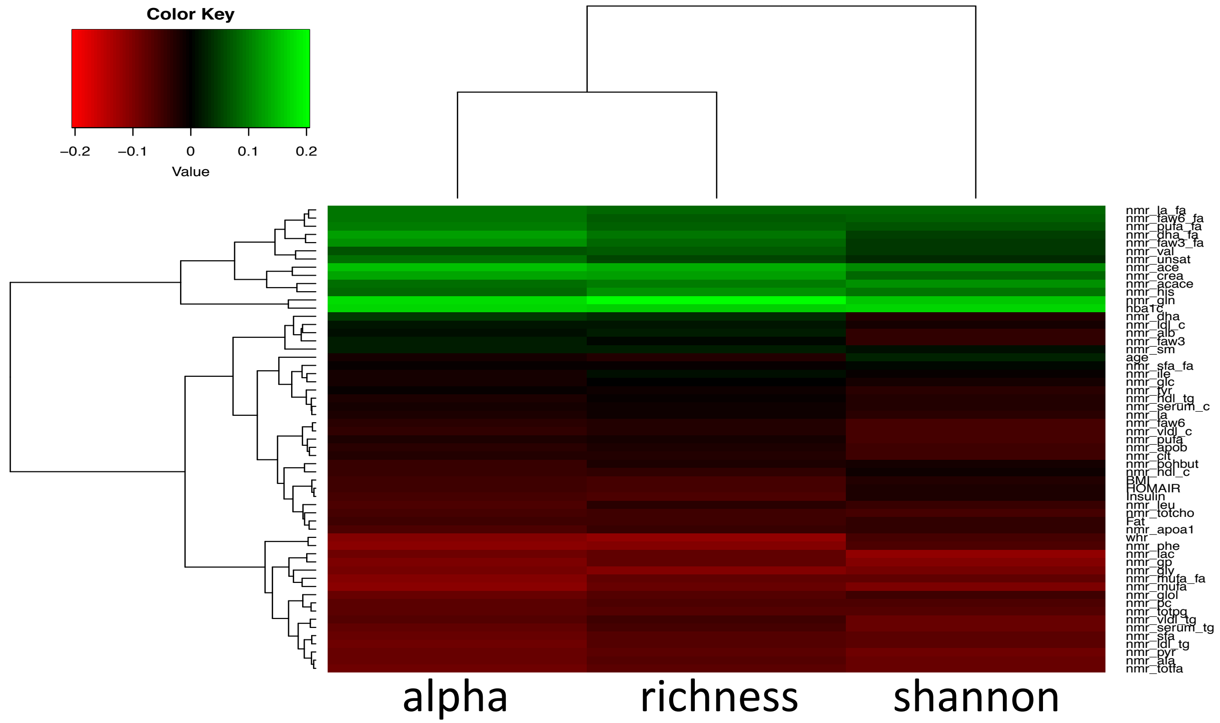

B.

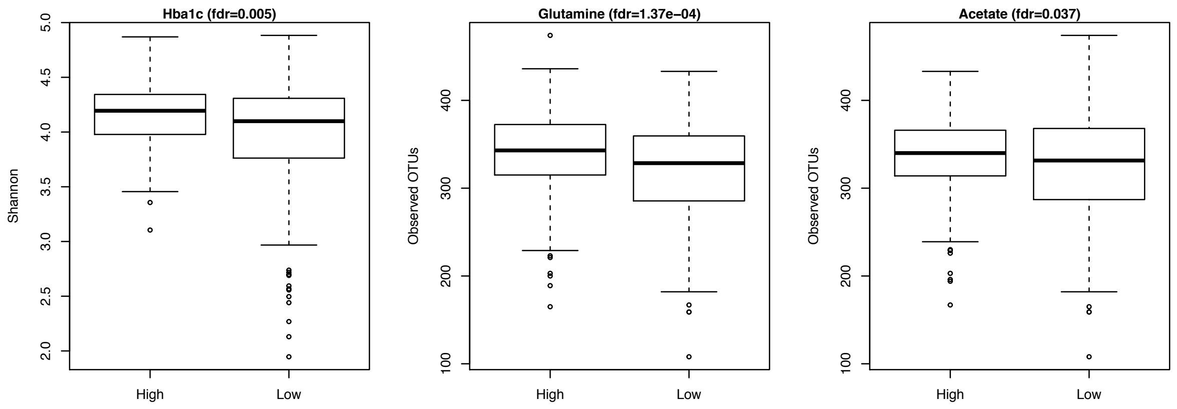

Figure S3: Associations of OTUs with fasting blood levels of metabolites. Significant associations of circulating serum metabolites with gut mirobiota OTUs (shared 50% of subjects) at FDR<0.05. Associations were adjusted for age and treatment.

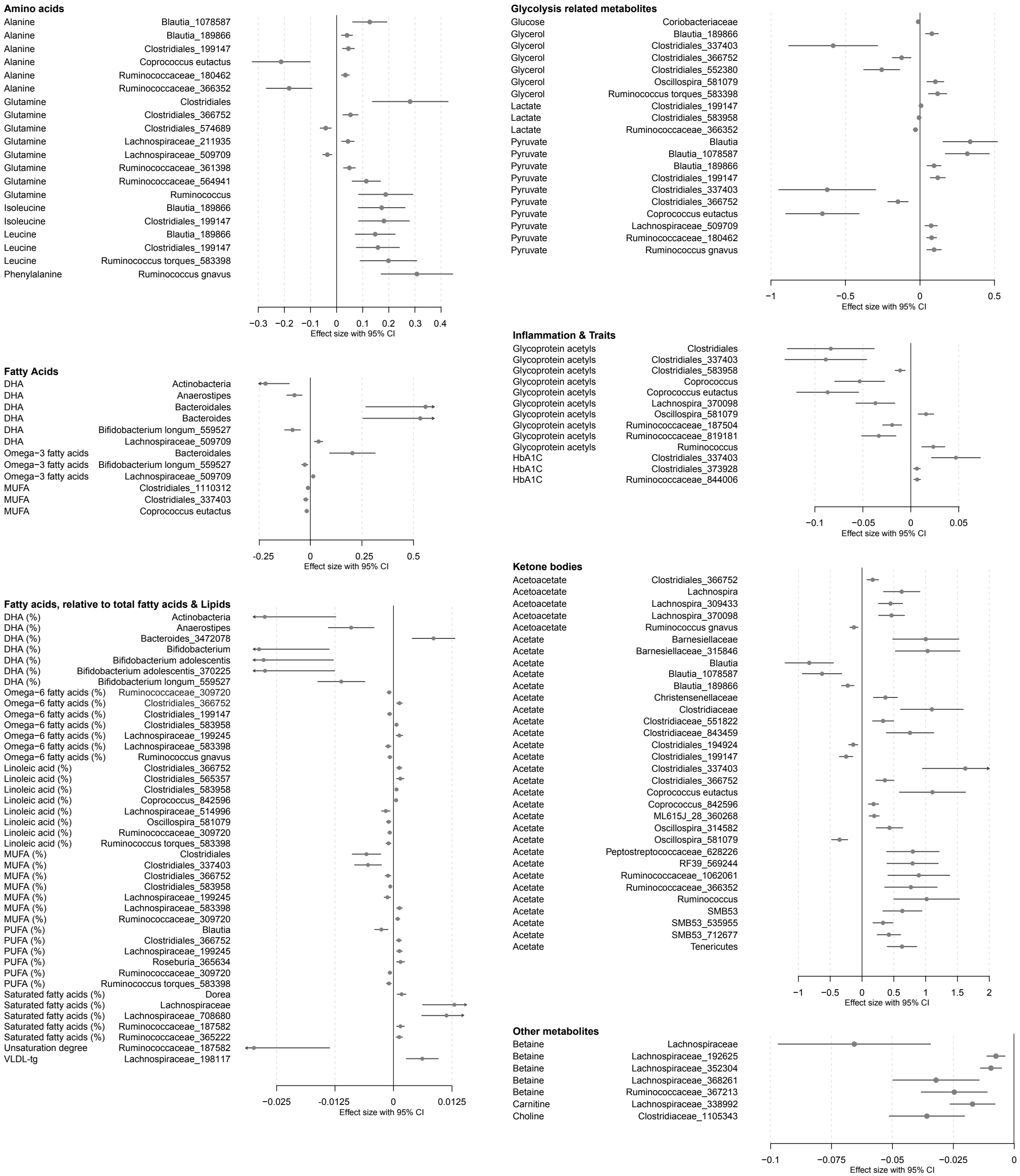

Figure S4: Boxplots of glutamine and branched chain amino acid (BCAA) levels in subjects with low and high BMI (A) and subjects in different HOMA-IR levels (B). *P* values were obtained using Tukey's HSD test of significance.

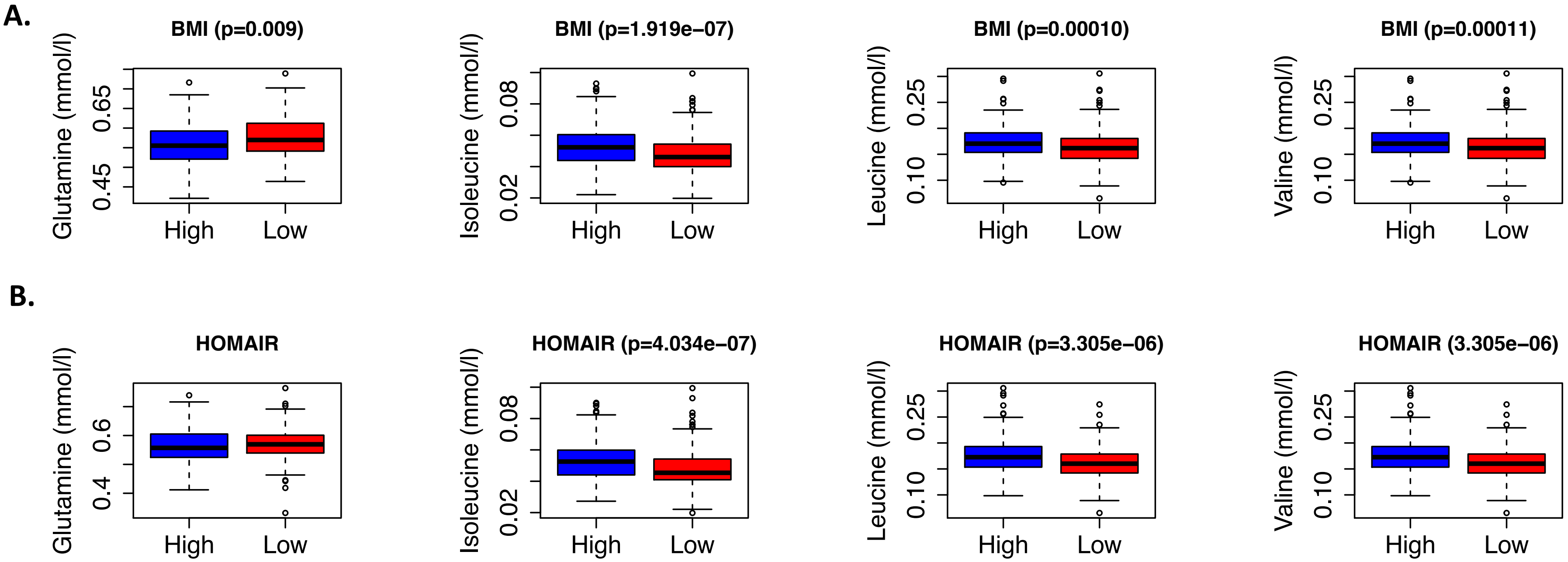

Figure S5: Correlation heatmap demonstrating the association between the metabolic traits and fasting TMAO concentrations. Coloring represents the Spearman correlation coefficient between TMAO and traits. FDRs are denoted: *, FDR < 0.01; * *, FDR < 0.001.

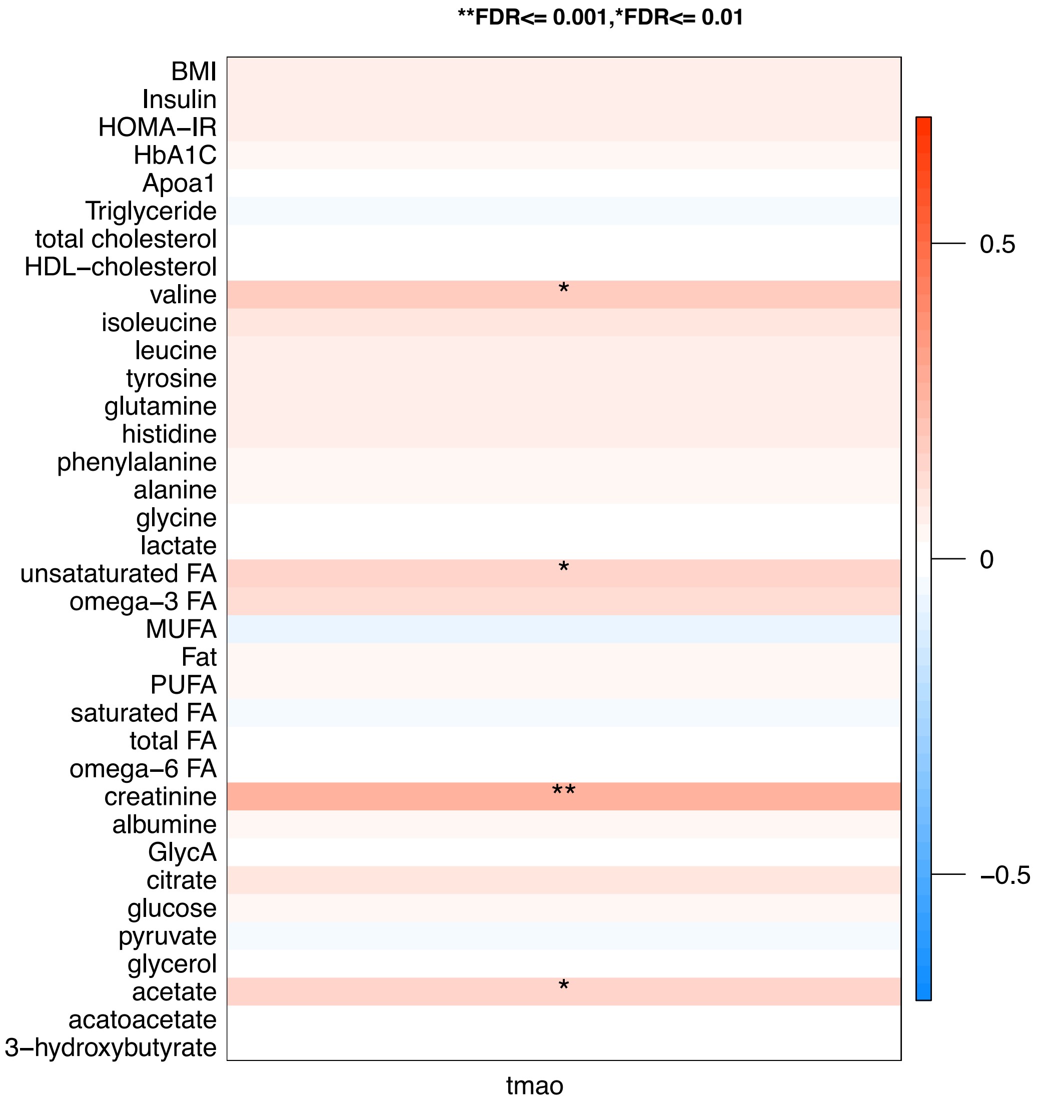

Figure S6: Gut microbiota profile in obese and type 2 diabetes. Boxplot of β diversity distances (unweighted UniFrac) between microbial communities (A), Ratio of Firmicutes to Bacteoridetes individuals in different BMI classes (BMI<25, BMI 25-30 and BMI>30 and between individuals based on glucose tolerance test (NGT, preT2D and newT2D) (B). Boxplot of HOMAIR and glycoprotein acetyls levels between individuals based on oral glucose tolerance test (OGTT) (C). * p< 0.01, **p< 0.001 for Tukey HSD test. OGTT descriptions see Methods.

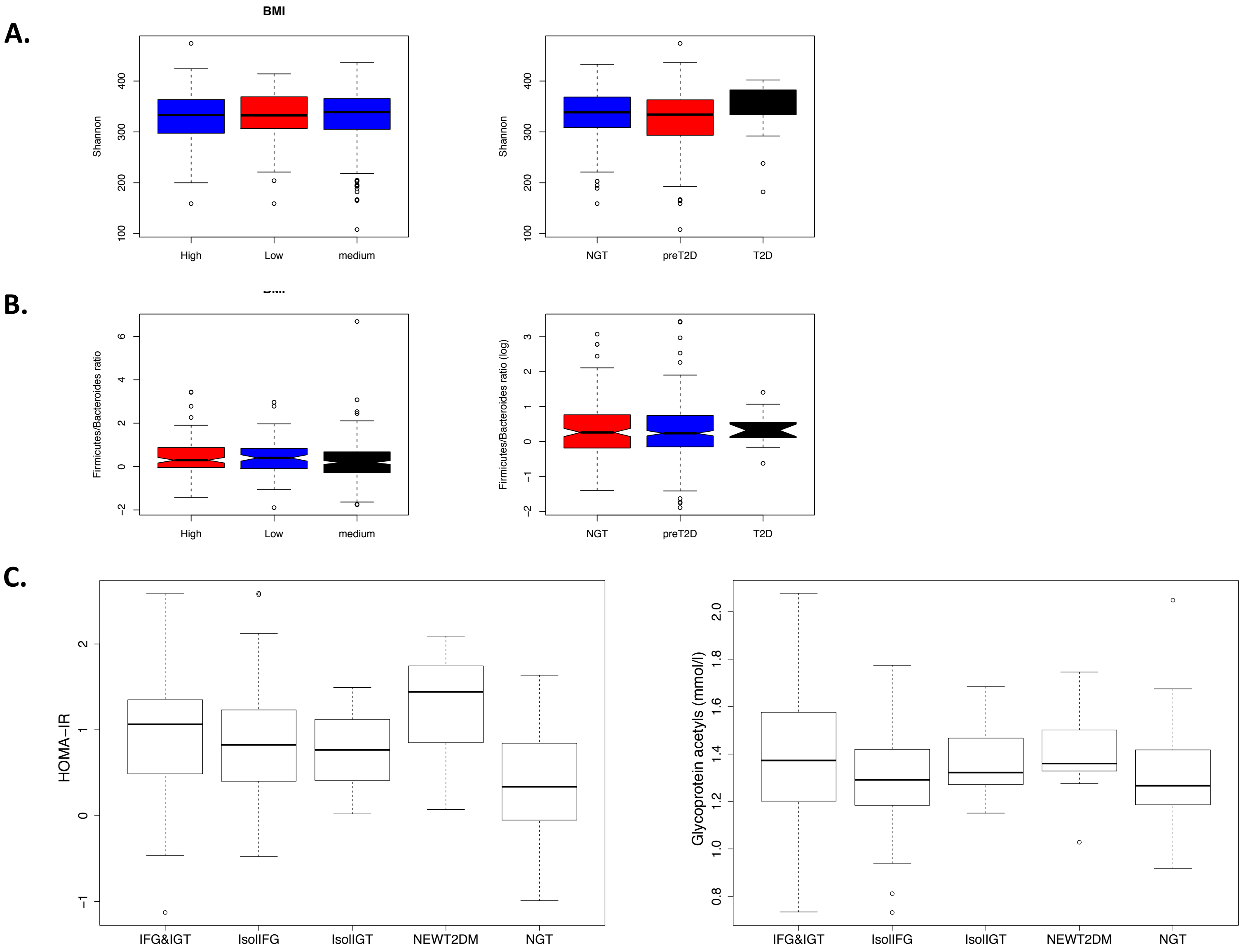

Figure S7: Comparison between preT2D and NGS OTU networks

A: OTU Co-occurrence networks for preT2D or NGS individuals. OTU (nodes) are colored by their corresponding WGCNA module colors. Edges are represented only for significant correlation between 2 OTU (P-value <0.01, abs (cor)>0.3). In both networks OTU are at the same locations. Network layout was based on OTU co-occurrence in preT2D condition.

B: Module-trait association: each cell of the matrix contains the correlation between one OTU module and a metabolic trait, and the corresponding p-value. The table is color-coded by correlation according to the color legend (red for positive correlations and green for negative correlations). Only traits associated with at least one module were represented (P-value<0.01).

C. Zsummary statistics of module preservation of preT2D OTU modules in NGS OTU modules (y-axis) vs. module size (x-axis). Green dashed line corresponds to the cutoff for very week preservation and the blue one to no preservation. D. Heatmap of the correlations between OTU abundance in tan module and glucose level in preT2D and NGS individuals. Circles sizes are proportional to correlation values. Scatterplot is represented for OTU 186090 in preT2D and NGS conditions.

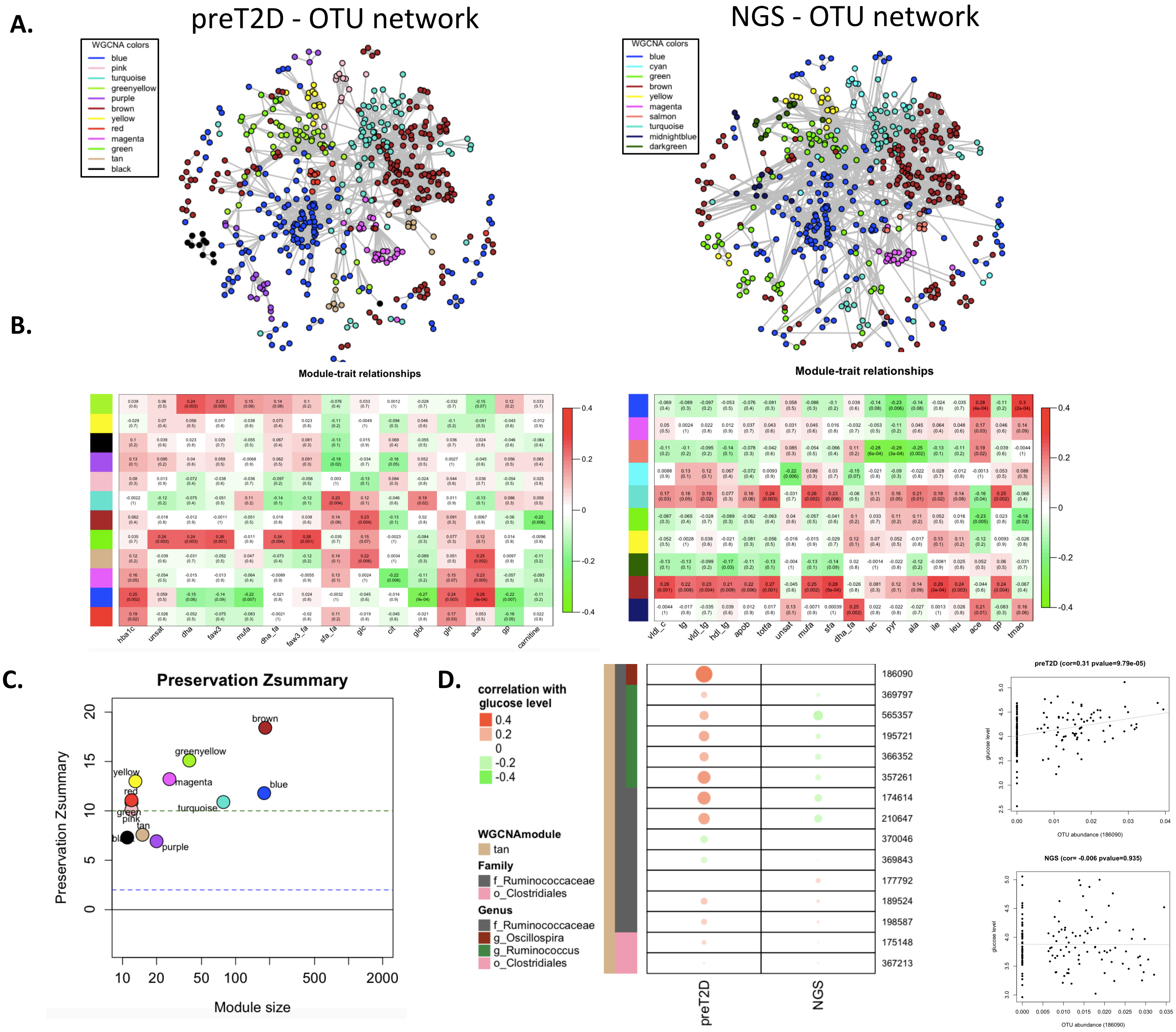
A.
preT2D - OTU network
WGCNA colors
blue
pink
turquoise
greenyellow
purple
brown
yellow
red
magenta
green
tan
black
NGS - OTU network
WGCNA colors
blue
cyan
green
brown
yellow
magenta
salmon
turquoise
midnightblue
darkgreen
B.
Module-trait relationships
hba1c
unsat
dha
faw3
mufa
dha_fa
faw3_fa
sfa_fa
glc
cit
glol
gln
ace
gp
carnitine
Module-trait relationships
vldl_c
tg
vldl_tg
hdl_tg
apob
totfa
unsat
mufa
sfa
dha_fa
lac
pyr
ala
ile
leu
ace
gp
tmao
C.
Preservation Zsummary
Preservation Zsummary
Module size
brown
greenyellow
yellow
magenta
blue
red
turquoise
green
pink
tan
black
purple
D.
correlation with glucose level
0.4
0.2
0
-0.2
-0.4
WGCNAmodule
tan
Family
f_Ruminococcaceae
o_Clostridiales
Genus
f_Ruminococcaceae
g_Oscillospira
g_Ruminococcus
o_Clostridiales
186090
369797
565357
195721
366352
357261
174614
210647
370046
369843
177792
189524
198587
175148
367213
preT2D
NGS
preT2D (cor=0.31 pvalue=9.79e-05)
glucose level
OTU abundance (186090)
NGS (cor= -0.006 pvalue=0.935)
glucose level
OTU abundance (186090)
